# Supplementary material for: Urinary metabolic biomarkers of diet quality in European children are associated with metabolic health
Source: eLife. 2022 Jan 25;11:e71332. doi: 10.7554/eLife.71332 (PMC8789316; doi:10.7554/eLife.71332)
Supplement: Supplementary file 1. — (a) KIDMED items, and their scoring, used to assess adherence to the Mediterranean diet in HELIX children. (b) Items included in the FFQ by ultra-processed food (UPF) inclusion and information regarding the extent and purpose of food processing. (c) Intakes of food groups (in servings/week) by categories of the KIDMED score. (d) Intakes of food groups (in servings/week) by quartiles of ultra-processed food intake. (e) Associations of KIDMED score with urinary metabolites in childhood. (f) Associations of ultra-processed food consumption with urinary metabolites in childhood. (g) Regression formulas (scores) for predicting diet quality in childhood based on panels of urinary metabolites. (h) Interaction of diet quality indicators in association to C-peptide concentration in childhood. (i) Associations of diet quality with C-peptide concentration in childhood after stratifying by sex and by weight status, respectively. (j) Associations between urinary metabolites linked to diet quality and C-peptide in childhood. [file elife-71332-supp1.docx]

[Supplementary file 1a. KIDMED items, and their scoring, used to assess adherence to the Mediterranean diet in HELIX children 2](#_Toc88665535)

[Supplementary file 1b. Items included in the FFQ by ultra-processed food (UPF) inclusion and information regarding the extent and purpose of food processing 3](#_Toc88665536)

[Supplementary file 1c. Intakes of food groups (in servings/week) by categories of the KIDMED score 5](#_Toc88665537)

[Supplementary file 1d. Intakes of food groups (in servings/week) by quartiles of ultra-processed food intake 6](#_Toc88665538)

[Supplementary file 1e. Associations of KIDMED score with urinary metabolites in childhood 7](#_Toc88665539)

[Supplementary file 1f. Associations of ultra-processed food consumption with urinary metabolites in childhood 9](#_Toc88665540)

[Supplementary file 1g. Regression formulas (scores) for predicting diet quality in childhood based on panels of urinary metabolites (creatinine-normalized concentrations, μmol/mmol of creatinine) 11](#_Toc88665541)

[Supplementary file 1h. Interaction of diet quality indicators in association to C-peptide concentration in childhood 12](#_Toc88665542)

[Supplementary file 1i. Associations of diet quality with C-peptide concentration in childhood after stratifying by sex and by weight status, respectively 13](#_Toc88665543)

[Supplementary file 1j. Associations between urinary metabolites (μmol/mmol of creatinine) linked to diet quality and C-peptide in childhood 14](#_Toc88665544)

# Supplementary file 1a. KIDMED items, and their scoring, used to assess adherence to the Mediterranean diet in HELIX children

| KIDMED items | Scoring | Overall prevalence (N=1147)^1^ | Prevalence by KIDMED categories (%)^2^ | | |
| --- | --- | --- | --- | --- | --- |
|  |  |  | Low (N=104) | Moderate (N=848) | High (N=195) |
| Takes a fruit or fruit juice every day | +1 | 60% | 17% | 57% | 94% |
| Has a second fruit every day | +1 | 0% | 0% | 0% | 0% |
| Has fresh or cooked vegetables regularly once a day | +1 | 35% | 7% | 29% | 77% |
| Has fresh or cooked vegetables more than once a day | +1 | 0% | 0% | 0% | 0% |
| Consumes fish regularly (at least 2 – 3 times per week) | +1 | 39% | 5% | 34% | 75% |
| Goes more than once a week to a fast-food (hamburger) restaurant | -1 | 12% | 38% | 10% | 3% |
| Likes pulses and eats them more than once a week | +1 | 28% | 8% | 25% | 49% |
| Consumes pasta or rice almost every day (5 or more time per week) | +1 | 0% | 0% | 0% | 0% |
| Consumes nuts regularly (at least 2 – 3 times per week) | +1 | 13% | 2% | 9% | 34% |
| Uses olive oil at home | +1 | 92% | 79% | 93% | 98% |
| Skips breakfast | -1 | 28% | 71% | 28% | 7% |
| Has a dairy product for breakfast (yoghurt, milk, etc.) | +1 | 95% | 83% | 95% | 99% |
| Has commercially baked goods or pastries for breakfast | -1 | 47% | 77% | 50% | 18% |
| Takes two yoghurts and/or some cheese (40 g) daily | +1 | 29% | 7% | 27% | 52% |
| Takes sweets and candy several times every day | -1 | 20% | 52% | 20% | 2% |
| ^1^ % answering positively to the question in the total study population of 1147 children. | | | | | |
| ^2^ % answering positively to the question within each of the KIDMED score categories, describing adherence levels of Mediterranean diet as: low, KIDMED score, <1; moderate, KIDMED score, 1-4; high, >4. | | | | | |

Supplementary file 1b. Items included in the FFQ by ultra-processed food (UPF) inclusion and information regarding the extent and purpose of food processing

| Food item in the FFQ^1^ | Description | Information on extent and purpose of food processing^2^ |
| --- | --- | --- |
| **Foods in the UPF group** | |  |
| Cookies | Plain biscuits and cookies | Assumption that most of these products are industrially made formulations of ingredients that result from a series of industrial processes, many requiring sophisticated equipment and technology. Such processes include the fractioning of whole foods into substances, chemical modifications of these substances, assembly of unmodified and modified food substances using industrial techniques such as extrusion, moulding and pre-frying, frequent application of additives whose function is to make the final product palatable or hyper-palatable (‘cosmetic additives’), and sophisticated packaging, usually with synthetic materials.  The used ingredients often include sugar, oils and fats, and salt, generally in combination; substances that are sources of energy and nutrients but of no or rare culinary use such as high fructose corn syrup, hydrogenated or interesterified oils, and protein isolates; cosmetic additives such as flavours, flavour enhancers, colours, emulsifiers, sweeteners, thickeners, and anti-foaming, bulking, carbonating. |
| Pastries | Buns, cakes, pastries, croissants, donuts, halva |  |
| Sugar-Sweetened Beverages | Sugar-sweetened soft/fizzy drinks (eg coca cola, fanta, ribena) |  |
| Artificially-sweetened and low-sugar beverages | Artificially sweetened/low sugar/sugar free soft drinks (eg. Diet fanta) |  |
| Cold meat cuts | Sausages, bacon, chorizo, salami (in slices) |  |
| Ham | Ham (in slices) |  |
| Dairy deserts | Dairy desert/ milky pudding, ice cream |  |
| Sugar sweetened breakfast cereals | Sugar-sweetened cereals for children |  |
| Other breakfast cereals | Other breakfast cereals/porridge |  |
| Crispbreads and rusks | Rusks, crispy bread, rice and corn cakes |  |
| Chocolate | Chocolate (bars, bonbon, spreads, cacao) |  |
| Sweets | Sweets, gummy bears |  |
| Margarine | Margarine |  |
| Dressings | Mayonnaise, ketchup, dressings |  |
| Salty snacks | Crisps, salty snacks, pipas |  |
| **Food not in the UPF group** | |  |
| Red meat | Red meat (beef, pork lamb, steak, burger, chop, meatballs, small curry, zeppelin meat dumpling, minced meat) | Assumption that most of these products are whole or in the form of steaks, fillets and other cuts, fresh or chilled or frozen. |
| Poultry | Poultry (chicken, turkey, burger, nuggets, curry) | Assumption that most of these products are whole or in the form of steaks, fillets and other cuts, fresh or chilled or frozen. |
| White fish | Lean/white fish (flat fish, perch, fish cakes) | Assumption that most of these products are whole or in the form of steaks, fillets and other cuts, fresh or chilled or frozen. |
| Oily fish | Fatty/oily fish (sardines, salmon, mackerel, tuna, trout, carp) | Assumption that most of these products are whole or in the form of steaks, fillets and other cuts, fresh or chilled or frozen. |
| Seafood | Seafood (shellfish, squids, shrimps) | Assumption that most of these products are whole or in the form of steaks, fillets and other cuts, fresh or chilled or frozen. |
| Canned fish | Canned fish (tuna, sardines, sprat) | Assumption that most of these products are processed in order to increase the durability of fresh foods and may contain additives that prolong product duration, protect original properties or prevent proliferation of microorganisms. |
| Egg | Egg (fried, scrambled, boiled, omelet) | Assumption that most of these products are made of fresh or chilled eggs. |

**Supplementary file 1b** - Continued

| **Food not in the UPF group** | |  |
| --- | --- | --- |
| Cheese | Cheese (eg cheddar, brie, feta, cures cheese, brown cheese, curd, cream cheese) | Assumption that most of these products are processed in order to increase the durability and make them more enjoyable by modifying or enhancing their sensory qualities. These products may contain additives that prolong product duration, protect original properties or prevent proliferation of microorganisms. |
| Milk | Milk (skimmed, semiskimmed, full fat milk) | Assumption that most of these products are not artificially flavored |
| Probiotic yoghurt | Pro-biotic yoghurt (eg. Actimel, kefir) | Assumption that most of these products are not artificially flavored |
| Yoghurt | Yoghurt (excluding pro-biotic) | Assumption that most of these products are not artificially flavored |
| Fruits | Fruits | Assumption that most of these products are fresh, squeezed, chilled, frozen fruits. |
| Canned fruit | Canned fruit with syrup/nectars | Assumption that most of these products are processed in order to increase the durability of fresh foods and may contain additives that prolong product duration, protect original properties or prevent proliferation of microorganisms. |
| Juice | Natural/pure/freshly squeezed fruit juice | Assumption that most of these products do not contain flavorings and sweeteners |
| Dry fruits | Dried fruit (raisins, currant, dry apricots, prunes, figs) | Assumption that most of these products are dried fruits. |
| Cooked vegetables | Cooked or pureed vegetables (no potatoes) | Assumption that most of these products are of fresh, squeezed, chilled, frozen, or dried leafy and root vegetables or from canned or bottled vegetables. |
| Potatoes | Potatoes (boiled, baked, mashed) not chips/fries | Assumption that most of these products are homemade from fresh potatoes. |
| Fries | French fries (1 portion) | Assumption that most of these products are homemade from fresh potatoes. |
| Vegetables | Raw vegetables | Assumption that most of these products are fresh, squeezed, chilled, frozen, or dried leafy and root vegetables. |
| Nuts | Nuts (almonds, walnuts) | Assumption that most of these products are nuts with or without salt or sugar. |
| Pulses | Pulses (chickpeas, lentils, peas, beans) | Assumption that most of these products are fresh or canned/bottled legumes. |
| Dark bread | Whole grain /dark bread/ ‘50/50” | Assumption that most of these products are fresh bread that contain different types of flour, water and yeast. |
| White bread | White bread | Assumption that most of these products are fresh bread that contain different types of flour, water and yeast. |
| Rice & pasta | Rise and pasta | Assumption that most of these products are grains and past made of flour. |
| Sugar and other sweeteners | Sugar, honey, jam | Assumption that most of these products are sugar from cane or beet, honey extracted from combs and jam made from fruit, sugar, water and other additives that prolong product duration. |
| Butter | Butter or ghee | Assumption that most of these products are from milk. |
| Olive oil | Olive oil for cooking or seasoning | Assumption that most of these products are vegetable oils crushed from olives. |
| Other oils | Other vegetable oils (canola oil) | Assumption that most of these products are vegetable oils crushed from seeds and nuts. |
| ^1^ Every item corresponds to one question in the FFQ. | | |
| ^2^ NOVA food groups: definition according to the extent and purpose of food processing from Monteiro CA et al. Ultra-processed foods: what they are and how to identify them. Public Health Nutrition (2019), Volume 22, Issue 5, pp. 936-94. | | |

Supplementary file 1c. Intakes of food groups (in servings/week) by categories of the KIDMED score**^1^**

|  | **KIDMED categories^2^** | | | |
| --- | --- | --- | --- | --- |
|  | **Low (N=104)** | **Moderate (N=848)** | **High (N=195)** | **P value^3^** |
| Vegetables | 4.5 (4.3) | 7.6 (5.7) | 13.5 (7.1) | <0.001 |
| Fruits | 6.3 (5.7) | 11.2 (7.9) | 16.8 (7.8) | <0.001 |
| Fish and seafood | 1.3 (0.9) | 2.5 (2.2) | 4 (2.7) | <0.001 |
| Meat | 8.1 (4.2) | 7.9 (4.5) | 8 (4.1) | 0.67 |
| Dairy products | 14.8 (8.7) | 21.1 (11.2) | 24.4 (12.1) | <0.001 |
| Pulses | 0.7 (0.9) | 1.2 (1.2) | 1.8 (1.3) | <0.001 |
| Bread & Cereals | 9.6 (8.2) | 11 (7.3) | 13.1 (7.1) | <0.001 |
| Eggs | 1.8 (1.5) | 1.8 (1.6) | 2 (1.8) | 0.33 |
| Nuts | 0.4 (0.5) | 0.7 (1.3) | 1.6 (1.9) | <0.001 |
| Potatoes | 3.8 (2.7) | 3.7 (2.7) | 3.7 (2.7) | 0.46 |
| Bakery products | 7.1 (5.5) | 5.3 (5) | 3.6 (3) | <0.001 |
| Added fats & oils | 2.4 (3.2) | 1.8 (2.9) | 1.5 (2.5) | 0.002 |
| Salty snacks | 1.7 (1.9) | 1.4 (2) | 1.4 (1.7) | 0.09 |
| Sweets | 11.6 (8.8) | 7.5 (6) | 6.7 (4.5) | <0.001 |
| Beverages | 2.8 (5.5) | 1.2 (3.1) | 1 (2.2) | 0.003 |
| Dressings | 1.7 (1.7) | 2.3 (2.8) | 1.9 (1.9) | 0.24 |

^1^ Values are mean (SD).

^2^ KIDMED score categories were defined as follows: low, <1; moderate, 1-4; high, >4.

^3^ P-value for differences across KIDMED score categories were derived using Kruskal-Wallis test.

Supplementary file 1d. Intakes of food groups (in servings/week) by quartiles of ultra-processed food intake**^1^**

|  | **Ultra-processed food intake^2^** | | | | |
| --- | --- | --- | --- | --- | --- |
|  | **Q1 (N=279)** | **Q2 (N=293)** | **Q3 (N=288)** | **Q4 (N=287)** | **P-value^3^** |
| Vegetables | 10.5 (7.2) | 9.1 (7) | 7.6 (5.1) | 6.0 (4.9) | <0.001 |
| Fruits | 14.3 (8.9) | 12.7 (7.7) | 11 (7.8) | 8.8 (7.2) | <0.001 |
| Fish and seafood | 3.4 (2.8) | 2.9 (2.5) | 2.4 (1.9) | 2 (1.8) | <0.001 |
| Meat | 7.3 (3.7) | 7.8 (4.1) | 8.1 (4.2) | 8.6 (5.5) | 0.03 |
| Dairy products | 22.6 (11.1) | 21.9 (12.2) | 20.4 (11) | 19.6 (11.1) | 0.004 |
| Pulses | 1.3 (1.2) | 1.3 (1.1) | 1.4 (1.2) | 1.2 (1.3) | 0.03 |
| Bread & Cereals | 13.2 (7.9) | 11.8 (7.3) | 10.4 (7) | 9.6 (7.1) | <0.001 |
| Eggs | 2 (1.9) | 1.9 (1.6) | 1.9 (1.3) | 1.5 (1.7) | <0.001 |
| Nuts | 1.1 (1.8) | 0.8 (1.2) | 0.7 (1.2) | 0.7 (1.5) | <0.001 |
| Potatoes | 3.4 (2.5) | 3.6 (2.8) | 3.9 (3.1) | 3.8 (2.4) | 0.02 |
| Bakery products | 2.9 (2.6) | 4.3 (3.3) | 5.5 (4.1) | 7.8 (6.9) | <0.001 |
| Added fats & oils | 1.7 (3.1) | 1.7 (2.7) | 1.7 (2.5) | 2 (3) | 0.01 |
| Salty snacks | 0.8 (0.9) | 1.1 (1.3) | 1.4 (1.9) | 2.3 (2.8) | <0.001 |
| Sweets | 5.9 (5) | 7 (5.1) | 7.7 (5.7) | 10.3 (7.7) | <0.001 |
| Beverages | 0.5 (0.9) | 0.9 (1.5) | 1.1 (2) | 2.8 (5.8) | <0.001 |
| Dressings | 1.3 (1.5) | 1.9 (2.3) | 2.2 (2.5) | 3.1 (3.5) | <0.001 |

^1^ Values are mean (SD).

^2^ Quartiles of ultra-processed food intake (expressed as % of total daily food intake) were as follows: Q1, ≤18%; Q2, >18% but <23%; Q3, ≥23% but <29%; and Q4, ≥29%.

^3^ P-value for differences across quartiles of ultra-processed food intake were derived using Kruskal-Wallis test.

# Supplementary file 1e. Associations of KIDMED score with urinary metabolites in childhood^1^

| **Urinary metabolite** | **Compound Class** | **Percent change (95% CI)** | **P-value** | **Q-value** |
| --- | --- | --- | --- | --- |
| *scyllo*-inositol | Alcohols and polyols | 0.23 (-4.98, 5.64) | 0.95 | 0.95 |
| *N*-methylnicotinic acid | Alkaloids and derivatives | 4.71 (1.67, 7.65) | <0.01 | 0.03 |
| *N*-methylpicolinic acid | Alkaloids and derivatives | 3.99 (-2.44, 10.71) | 0.23 | 0.48 |
| 2-hydroxyisobutyrate | Alpha hydroxy acids and derivatives | 1.16 (-0.15, 2.28) | 0.09 | 0.29 |
| Lactate^2^ | Alpha hydroxy acids and derivatives | -0.92 (-2.96, 1.15) | 0.38 | 0.63 |
| Dimethylamine | Amines | 1.86 (-0.91, 4.55) | 0.2 | 0.44 |
| Trimethylamine | Amines | 2.09 (0.33, 3.67) | 0.02 | 0.11 |
| 3-aminoisobutyrate | Amino acids, peptides, and analogues | 1.39 (-2.1, 4.85) | 0.45 | 0.67 |
| 5-oxoproline | Amino acids, peptides, and analogues | 0.23 (-2.12, 2.72) | 0.83 | 0.89 |
| Alanine | Amino acids, peptides, and analogues | -1.37 (-2.86, 0.23) | 0.1 | 0.29 |
| Creatine | Amino acids, peptides, and analogues | 0.93 (-3.03, 5.08) | 0.65 | 0.84 |
| Glutamine | Amino acids, peptides, and analogues | -0.92 (-4.15, 2.27) | 0.55 | 0.73 |
| Glycine | Amino acids, peptides, and analogues | -1.14 (-2.93, 0.71) | 0.23 | 0.48 |
| Isoleucine | Amino acids, peptides, and analogues | 2.09 (-0.86, 5.18) | 0.17 | 0.41 |
| Leucine | Amino acids, peptides, and analogues | 0.23 (-0.93, 1.47) | 0.67 | 0.84 |
| Lysine | Amino acids, peptides, and analogues | -0.23 (-2.59, 2.06) | 0.81 | 0.89 |
| Proline betaine | Amino acids, peptides, and analogues | 6.66 (-2.04, 16.05) | 0.14 | 0.37 |
| Tyrosine | Amino acids, peptides, and analogues | 2.33 (0.29, 4.2) | 0.02 | 0.13 |
| Valine | Amino acids, peptides, and analogues | 0.69 (-0.48, 1.72) | 0.27 | 0.51 |
| 3-hydroxybutyrate/3-aminoisobutyrate^3^ | Amino acids, peptides, and analogues | 0.93 (-4.14, 6.16) | 0.74 | 0.88 |
| Trimethylamine *N*-oxide | Aminoxides | 5.44 (-0.31, 11.66) | 0.06 | 0.23 |
| 3-Indoxylsulfate | Arylsulfates | 3.51 (-0.07, 7.07) | 0.06 | 0.21 |
| *p*-cresol sulfate | Arylsulfates | 3.28 (0.04, 6.83) | 0.05 | 0.21 |
| Hippurate | Benzoic acids and derivatives | 5.93 (3.39, 8.55) | <0.001 | <0.001 |
| 3-hydroxyisobutyrate | Beta hydroxy acids and derivatives | -0.46 (-2.24, 1.16) | 0.53 | 0.73 |
| Glucose | Carbohydrates and carbohydrate conjugates | -0.23 (-1.94, 1.58) | 0.83 | 0.89 |
| *N*-acetyl neuraminic acid | Carbohydrates and carbohydrate conjugates | 0.46 (-2.68, 3.64) | 0.79 | 0.89 |
| Sucrose | Carbohydrates and carbohydrate conjugates | -5.81 (-9.87, -1.67) | 0.01 | 0.05 |
| Acetate | Carboxylic acids | 3.51 (0.95, 6.07) | 0.01 | 0.05 |
| Formate | Carboxylic acids | 1.86 (-0.38, 3.94) | 0.11 | 0.31 |
| Carnitine^4^ | Carnitines | -2.28 (-6.45, 1.97) | 0.28 | 0.51 |

**Supplementary file 1e** - Continued

| **Urinary metabolite** | **Compound Class** | **Percent change (95% CI)** | **P value** | **Q value** |
| --- | --- | --- | --- | --- |
| Succinate | Dicarboxylic acids and derivatives | 3.51 (-3.61, 11.28) | 0.34 | 0.58 |
| 3-hydroxyisovalerate | Hydroxy fatty acids | 0.23 (-1.2, 1.85) | 0.69 | 0.84 |
| Acetone | Ketones | 2.33 (-0.98, 5.6) | 0.17 | 0.41 |
| *N*1-methyl-nicotinamide | Nicotinamide | 1.16 (-1.68, 4.23) | 0.41 | 0.63 |
| *N*-methyl-2-pyridone-5-carboxamide | Nicotinamides | 0.46 (-4.73, 5.98) | 0.86 | 0.9 |
| Urea | Organic carbonic acids and derivatives | 2.33 (1.04, 3.47) | <0.001 | 0.01 |
| Taurine | Organosulfonic acids | 1.62 (-2.84, 6.51) | 0.46 | 0.67 |
| *p*-hydroxyphenylacetate | Phenols | 1.62 (0, 3.07) | 0.05 | 0.21 |
| Pantothenic acid | Polyols | 1.62 (0.55, 2.55) | <0.01 | 0.03 |
| 4-deoxyerythronic acid | Sugar acids and derivatives | 0.93 (-0.68, 2.53) | 0.26 | 0.51 |
| 4-deoxythreonic acid | Sugar acids and derivatives | 0.69 (-1.04, 2.59) | 0.41 | 0.63 |
| Citrate | Tricarboxylic acids and derivatives | 0.00 (-2.23, 2.47) | 0.94 | 0.95 |

Abbreviations: KIDMED score, Mediterranean Diet Quality Index for children and adolescents.

^1^ Effect estimates are percent changes (95% CI) in metabolite levels (in log10 μmol/mmol of creatinine) per 1-unit increase in the KIDMED score and were derived from linear regression models adjusted for maternal age, maternal education level, maternal pre-pregnancy BMI, family affluence status, child sex, child age, child BMI, child sedentary behavior, child ethnicity, and a cohort indicator.

^2^ Threonine could also contribute as a minor component in the signal for lactate

^3^ Overlapping signal between 3-hydroxybutyrate/3-aminoisobutyrate

^4^ Choline could also contribute as a minor component in the signal of carnitine

#

# Supplementary file 1f. Associations of ultra-processed food consumption with urinary metabolites in childhood**^1^**

| **Urinary metabolite** | **Compound Class** | **Percent change (95% CI)** | **P-value** | **Q-value** |
| --- | --- | --- | --- | --- |
| *scyllo*-inositol | Alcohols and polyols | 0.69 (-4.46, 5.98) | 0.81 | 0.85 |
| *N*-methylnicotinic acid | Alkaloids and derivatives | -3.84 (-6.59, -1.22) | 0.01 | 0.04 |
| *N*-methylpicolinic acid | Alkaloids and derivatives | -1.83 (-7.71, 4.45) | 0.56 | 0.71 |
| 2-hydroxyisobutyrate | Alpha hydroxy acids and derivatives | -1.6 (-2.65, -0.33) | 0.01 | 0.07 |
| Lactate^2^ | Alpha hydroxy acids and derivatives | -0.92 (-3, 1.03) | 0.33 | 0.53 |
| Dimethylamine | Amines | 0.69 (-1.99, 3.29) | 0.65 | 0.77 |
| Trimethylamine | Amines | -0.46 (-1.97, 1.23) | 0.64 | 0.77 |
| 3-aminoisobutyrate | Amino acids, peptides, and analogues | -3.17 (-6.32, 0.17) | 0.06 | 0.23 |
| 3-hydroxybutyrate/3-aminoisobutyrate^3^ | Amino acids, peptides, and analogues | -2.95 (-7.69, 1.99) | 0.24 | 0.42 |
| 5-oxoproline | Amino acids, peptides, and analogues | -1.83 (-4.15, 0.47) | 0.12 | 0.26 |
| Alanine | Amino acids, peptides, and analogues | -0.46 (-2.06, 0.99) | 0.48 | 0.68 |
| Creatine | Amino acids, peptides, and analogues | -0.92 (-4.68, 3.12) | 0.67 | 0.78 |
| Glutamine | Amino acids, peptides, and analogues | -0.69 (-3.71, 2.6) | 0.71 | 0.78 |
| Glycine | Amino acids, peptides, and analogues | 0 (-1.8, 1.81) | 0.99 | 0.99 |
| Isoleucine | Amino acids, peptides, and analogues | -2.5 (-5.21, 0.44) | 0.10 | 0.26 |
| Leucine | Amino acids, peptides, and analogues | -1.37 (-2.51, -0.2) | 0.02 | 0.09 |
| Lysine | Amino acids, peptides, and analogues | 0 (-2.34, 2.21) | 0.94 | 0.96 |
| Proline betaine | Amino acids, peptides, and analogues | -2.95 (-10.61, 5.53) | 0.49 | 0.68 |
| Tyrosine | Amino acids, peptides, and analogues | -2.95 (-4.67, -1.03) | 0.002 | 0.03 |
| Valine | Amino acids, peptides, and analogues | -1.6 (-2.62, -0.51) | 0.004 | 0.03 |
| Trimethylamine *N*-oxide | Aminoxides | -1.83 (-7.18, 3.73) | 0.50 | 0.68 |
| 3-Indoxylsulfate | Arylsulfates | -2.28 (-5.53, 1.07) | 0.18 | 0.35 |
| *p*-cresol sulfate | Arylsulfates | -2.95 (-6, 0.23) | 0.07 | 0.23 |
| Hippurate | Benzoic acids and derivatives | -5.59 (-7.78, -3.28) | <0.001 | <0.001 |
| 3-hydroxyisobutyrate | Beta hydroxy acids and derivatives | -1.37 (-2.96, 0.34) | 0.12 | 0.26 |
| Glucose | Carbohydrates and carbohydrate conjugates | -1.14 (-2.84, 0.57) | 0.19 | 0.35 |
| *N*-acetyl neuraminic acid | Carbohydrates and carbohydrate conjugates | 0.69 (-2.43, 3.77) | 0.69 | 0.78 |
| Sucrose | Carbohydrates and carbohydrate conjugates | 8.39 (3.9, 13.1) | <0.001 | 0.003 |
| Acetate | Carboxylic acids | -1.83 (-4.1, 0.66) | 0.15 | 0.32 |
| Formate | Carboxylic acids | -1.83 (-3.83, 0.26) | 0.09 | 0.26 |
| Carnitine^4^ | Carnitines | -2.28 (-6.3, 1.95) | 0.29 | 0.48 |

**Supplementary file 1f** - Continued

| **Urinary metabolite** | **Compound Class** | **Percent change (95% CI)** | **P-value** | **Q-value** |
| --- | --- | --- | --- | --- |
| Succinate | Dicarboxylic acids and derivatives | -2.73 (-9.33, 4.35) | 0.44 | 0.65 |
| 3-hydroxyisovalerate | Hydroxy fatty acids | -1.6 (-3.08, -0.17) | 0.03 | 0.11 |
| Acetone | Ketones | -2.5 (-5.53, 0.6) | 0.11 | 0.26 |
| *N*1-methyl-nicotinamide | Nicotinamide | -1.14 (-3.95, 1.71) | 0.42 | 0.65 |
| *N*-methyl-2-pyridone-5-carboxamide | Nicotinamides | 0.69 (-4.35, 6.16) | 0.77 | 0.83 |
| Urea | Organic carbonic acids and derivatives | -2.73 (-3.76, -1.5) | <0.001 | <0.001 |
| Taurine | Organosulfonic acids | -5.38 (-9.52, -1.03) | 0.02 | 0.08 |
| *p*-hydroxyphenylacetate | Phenols | -0.46 (-1.91, 1.04) | 0.55 | 0.71 |
| Pantothenic acid | Polyols | -0.92 (-1.78, 0.15) | 0.10 | 0.26 |
| 4-deoxyerythronic acid | Sugar acids and derivatives | -0.92 (-2.38, 0.7) | 0.28 | 0.48 |
| 4-deoxythreonic acid | Sugar acids and derivatives | -2.05 (-3.84, -0.41) | 0.02 | 0.08 |
| Citrate | Tricarboxylic acids and derivatives | -1.83 (-4.05, 0.46) | 0.12 | 0.26 |

Abbreviations: UPF, ultra-processed food intake.

^1^ Effect estimates are percent changes (95% CI) in metabolite levels (in log10 μmol/mmol of creatinine) per 5% increase in daily UPF intake and were derived from linear regression models adjusted for maternal age, maternal education level, maternal pre-pregnancy BMI, family affluence status, child sex, child age, child BMI, child sedentary behavior, child ethnicity, and a cohort indicator.

^2^ Threonine could also contribute as a minor component in the signal for lactate

^3^ Overlapping signal between 3-hydroxybutyrate/3-aminoisobutyrate

^4^ Choline could also contribute as a minor component in the signal of carnitine

# Supplementary file 1g. Regression formulas (scores) for predicting diet quality in childhood based on panels of urinary metabolites (creatinine-normalized concentrations, μmol/mmol of creatinine)

|  | **KIDMED** | **UPF** |
| --- | --- | --- |
| Panel of 4 common metabolites linked to both diet quality indicators | -16.67 + 2.28*Hippurate (log10 μmol/mmol) -1.66*Sucrose (log10 μmol/mmol) + 4.18*Urea (log10 μmol/mmol) + 0.67*N-methylnicotinic acid (log10 μmol/mmol) | 10.36 - 1.11*Hippurate (log10 μmol/mmol) + 0.98*Sucrose (log10 μmol/mmol) - 2.81*Urea (log10 μmol/mmol) - 0.53*N-methylnicotinic acid (log10 μmol/mmol) |
| Panel of 4 common metabolites plus metabolites linked to KIDMED only | -13.46 + 2.00*Hippurate (log10 μmol/mmol) -1.22*Sucrose (log10 μmol/mmol) + 3.23*Urea (log10 μmol/mmol) + 0.32*N-methylnicotinic acid (log10 μmol/mmol) - 0.23*Pantothenic Acid (log10 μmol/mmol) + 0.22*Acetate (log10 μmol/mmol) | - |
| Panel of 4 common metabolites plus metabolites linked to UPF only | - | 9.10 - 1.12*Hippurate (log10 μmol/mmol) + 1.00*Sucrose (log10 μmol/mmol) – 2.15*Urea (log10 μmol/mmol) – 0.50*N-methylnicotinic acid (log10 μmol/mmol) - 0.41*Valine (log10 μmol/mmol) - 0.83*Tyrosine (log10 μmol/mmol) |

Abbreviations: KIDMED, Mediterranean Diet Quality Index for children and adolescents; UPF, ultra-processed food

# Supplementary file 1h. Interaction of diet quality indicators in association to C-peptide concentration in childhood^1^

|  | **C-peptide** |
| --- | --- |
|  | **Percent change (95% CI)** |
| KIDMED score (UPF intake, % of total daily food intake)^2^ |  |
| KIDMED ≤4, UPF <23% | *Ref.* |
| KIDMED ≤4, UPF ≥23% | 20.9 (-3.9, 52.0) |
| KIDMED >4, UPF <23% | -16.2 (-39.8, 16.5) |
| KIDMED >4, UPF ≥23% | 21.3 (-29.6, 109.1) |
| P value for interaction | 0.57 |

Abbreviations: KIDMED, Mediterranean Diet Quality Index for children and adolescents; UPF, ultra-processed food

^1^ Effect estimates represent percent changes in log-2 transformed C-peptide levels and their 95% CIs per combination of the KIDMED score and median-based UPF intake and were derived from linear regression models adjusted for maternal age, maternal education level, maternal pre-pregnancy BMI, family affluence status, child sex, child age, child BMI, child sedentary behavior, child ethnicity, postprandial interval, and a cohort indicator.

^2^ The following number of children were included for each combination of the KIDMED score and UPF intake: 422 children with low/moderate KIDMED score (≤4) and UPF intake below the median population intake (<23%); 530 children with low/moderate KIDMED score and UPF intake above the median population intake (≥23%); 150 children with high KIDMED score and UPF intake below the median population intake (≥23%); and 45 children with high KIDMED score and UPF intake above the median population intake.

# Supplementary file 1i. Associations of diet quality with C-peptide concentration in childhood after stratifying by sex and by weight status, respectively^1^

|  | **KIDMED score** | **UPF intake** |
| --- | --- | --- |
| **Sex** |  |  |
| Boys (N=626) | -7.5 (-14.8, 0.5) | 7.5 (-0.8, 16.5) |
| Girls (N=521) | -8.8 (-16.4, -0.5) | 11.5 (2.1, 21.8) |
| P-interaction | 0.80 | 0.53 |
| **Weight status^2^** |  |  |
| Normal weight (N=906) | -9.5 (-15.5, -3.0) | 10.5 (3.2, 18.4) |
| Overweight/Obese (N=237) | -4.5 (-16.7, 9.5) | 4.6 (-7.1, 18.9) |
| P-interaction | 0.48 | 0.45 |

Abbreviations: KIDMED, Mediterranean Diet Quality Index for children and adolescents; UPF, ultra-processed food

^1^ Effect estimates represent percent changes in log-2 transformed C-peptide levels and their 95% CIs per 1-unit increase in the KIDMED score or per 5% increase in daily UPF intake and were derived from linear regression models adjusted for maternal age, maternal education level, maternal pre-pregnancy BMI, family affluence status, child sex (for models not stratified by sex), child age, child BMI (for models not stratified by weight status), child sedentary behavior, child ethnicity, postprandial interval, and a cohort indicator.

^2^ Categories of normal weight and overweight/obese were derived using the International Obesity Taskforce criteria.

# Supplementary file 1j. Associations between urinary metabolites (μmol/mmol of creatinine) linked to diet quality and C-peptide in childhood^1^

|  | **C-peptide** |
| --- | --- |
| **Metabolites scores based on panels of metabolites, per SD** |  |
| Score for KIDMED based on the 4 metabolites linked to both KIDMED and UPF^2^ | -11.72 (-20.19, -2.34) |
| Score for KIDMED based on the 4 metabolites linked to both KIDMED and UPF plus metabolites linked to KIDMED only^3^ | -11.91 (-20.36, -2.55) |
| Score for UPF based on the 4 metabolites linked to both KIDMED and UPF^4^ | 11.90 (1.15, 23.79) |
| Score for UPF based on the 4 metabolites linked to both KIDMED and UPF plus metabolites linked to UPF only ^5^ | 11.66 (0.93, 23.52) |
| **Individual urinary metabolites, per log10 μmol/mmol** | **Percent change (95% CI)** |
| Acetate | 21 (-14, 70.1) |
| Hippurate | -13.2 (-38.4, 22.3) |
| *N*-methylnicotinic acid | 7.8 (-19.7, 44.7) |
| Pantothenic acid | 25.8 (-46.3, 194.8) |
| Sucrose | 24.7 (2.8, 51.3) |
| Tyrosine | -7.6 (-40.4, 43.5) |
| Urea | 17.5 (-42, 138.4) |
| Valine | 31.9 (-38.9, 185.1) |

Abbreviations: KIDMED, Mediterranean Diet Quality Index for children and adolescents; UPF, ultra-processed food

^1^ Effect estimates derived from linear regression models represent percent changes in log-2 transformed C-peptide levels and their 95% CIs per log10 μmol/mmol increase in individual metabolite concentrations and SD increase in metabolite scores.

^2^ Score= -16.67 + 2.28*Hippurate (log10 μmol/mmol) -1.66*Sucrose (log10 μmol/mmol) + 4.18*Urea (log10 μmol/mmol) + 0.67*N-methylnicotinic acid (log10 μmol/mmol)

^3^ Score= -13.46 + 2.00*Hippurate (log10 μmol/mmol) -1.22*Sucrose (log10 μmol/mmol) + 3.23*Urea (log10 μmol/mmol) + 0.32*N-methylnicotinic acid (log10 μmol/mmol) - 0.23*Pantothenic Acid (log10 μmol/mmol) + 0.22*Acetate (log10 μmol/mmol)

^4^ Score= 10.36 - 1.11*Hippurate (log10 μmol/mmol) + 0.98*Sucrose (log10 μmol/mmol) - 2.81*Urea (log10 μmol/mmol) - 0.53*N-methylnicotinic acid (log10 μmol/mmol)

^5^ Score= 9.10 - 1.12*Hippurate (log10 μmol/mmol) + 1.00*Sucrose (log10 μmol/mmol) – 2.15*Urea (log10 μmol/mmol) – 0.50*N-methylnicotinic acid (log10 μmol/mmol) - 0.41*Valine (log10 μmol/mmol) - 0.83*Tyrosine (log10 μmol/mmol)
