# Supplementary material for: Urinary metabolic biomarkers of diet quality in European children are associated with metabolic health
Source: eLife. 2022 Jan 25;11:e71332. doi: 10.7554/eLife.71332 (PMC8789316; doi:10.7554/eLife.71332)
Supplement: Transparent reporting form [file elife-71332-transrepform1.docx]

***eLife’s* transparent reporting form**

We encourage authors to provide detailed information *within their submission* to facilitate the interpretation and replication of experiments. Authors can upload supporting documentation to indicate the use of appropriate reporting guidelines for health-related research (see [EQUATOR Network](http://www.equator-network.org/%20)), life science research (see the [BioSharing Information Resource](https://biosharing.org/)), or the [ARRIVE guidelines](http://www.plosbiology.org/article/info:doi/10.1371/journal.pbio.1000412) for reporting work involving animal research. Where applicable, authors should refer to any relevant reporting standards documents in this form.

If you have any questions, please consult our Journal Policies and/or contact us: [editorial@elifesciences.org](mailto:editorial@elifesciences.org).

**Sample-size estimation**

- You should state whether an appropriate sample size was computed when the study was being designed
- You should state the statistical method of sample size computation and any required assumptions
- If no explicit power analysis was used, you should describe how you decided what sample (replicate) size (number) to use

Please outline where this information can be found within the submission (e.g., sections or figure legends), or explain why this information doesn’t apply to your submission:

To our knowledge, this study is one of the largest studies conducted to date examining diet quality, urinary metabolomics and metabolic health in a pediatric population. No power analysis was used. Sample size was based on the availability of data of interest in the HELIX cohort, as described in the Methods section under Study Population. Figure 1 provides the participant flowchart.

**Replicates**

- You should report how often each experiment was performed
- You should include a definition of biological versus technical replication
- The data obtained should be provided and sufficient information should be provided to indicate the number of independent biological and/or technical replicates
- If you encountered any outliers, you should describe how these were handled
- Criteria for exclusion/inclusion of data should be clearly stated
- High-throughput sequence data should be uploaded before submission, with a private link for reviewers provided (these are available from both GEO and ArrayExpress)

Please outline where this information can be found within the submission (e.g., sections or figure legends), or explain why this information doesn’t apply to your submission:

This is an observational study, not including experiments. Data used for this study were collected through questionnaires and measurements in biological samples.

Details of participants and data included in the analysis is clearly described in the Methods section under Study population. Inclusion/exclusion criteria are provided in the Methods Section under Study population and Figure 1. Outliers were not excluded from analysis.

Further information on the dietary background of participants and how the diet quality indicators were derived is provided in the Supplementary File 1.

**Statistical reporting**

- Statistical analysis methods should be described and justified
- Raw data should be presented in figures whenever informative to do so (typically when N per group is less than 10)
- For each experiment, you should identify the statistical tests used, exact values of N, definitions of center, methods of multiple test correction, and dispersion and precision measures (e.g., mean, median, SD, SEM, confidence intervals; and, for the major substantive results, a measure of effect size (e.g., Pearson's r, Cohen's d)
- Report exact p-values wherever possible alongside the summary statistics and 95% confidence intervals. These should be reported for all key questions and not only when the p-value is less than 0.05.

Please outline where this information can be found within the submission (e.g., sections or figure legends), or explain why this information doesn’t apply to your submission:

Statistical analysis is described in detail and justified in the methods section under Statistical analysis.

Raw data are presented in Table 1, Supplementary Files 1a-1d , and Figure 2.

Exact values on N and dispersion and precision measures of characteristics are given in Methods under Study Population and Table 1. Statistical tests conducted are described in the Methods section under Statistical analysis and in the legend of Tables and Figure presenting statistical analysis results. Precision measures (95% CIs) and P-values of effect estimates are provided in the Tables and Figures presenting statistical analysis results.

For the metabolomic analyses, a large number of statistical tests were run, and so we present Q values along with the P values of each test (Supplementary Files 1e and 1f).

(For large datasets, or papers with a very large number of statistical tests, you may upload a single table file with tests, Ns, etc., with reference to sections in the manuscript.)

**Group allocation**

- Indicate how samples were allocated into experimental groups (in the case of clinical studies, please specify allocation to treatment method); if randomization was used, please also state if restricted randomization was applied
- Indicate if masking was used during group allocation, data collection and/or data analysis

Please outline where this information can be found within the submission (e.g., sections or figure legends), or explain why this information doesn’t apply to your submission:

This is not applicable to our study. Participants were categorized into the different diet quality categories based on their dietary intake as reported in the food frequency questionnaires.

**Additional data files (“source data”)**

- We encourage you to upload relevant additional data files, such as numerical data that are represented as a graph in a figure, or as a summary table
- Where provided, these should be in the most useful format, and they can be uploaded as “Source data” files linked to a main figure or table
- Include model definition files including the full list of parameters used
- Include code used for data analysis (e.g., R, MatLab)
- Avoid stating that data files are “available upon request”

Please indicate the figures or tables for which source data files have been provided:

Source data for Figure 3 are provided in Supplementary Files 1e and 1f. Regression formulas of Figure 4 are provided in the Supplementary file 1g. Code used for data analysis has been clearly described with references in the methods section under Statistical analysis. The full description of parameters used is listed in Methods under Dietary assessment, Urine metabolite profiling, Plasma C-peptide, and Covariates.
